# Aging diabetes, deconstructing the cerebrovascular wall

**DOI:** 10.18632/aging.202963

**Published:** 2021-04-12

**Authors:** Fan Fan, George W. Booz, Richard J. Roman

**Affiliations:** 1 Department of Pharmacology and Toxicology, University of Mississippi Medical Center, Jackson, MS 39216, USA

**Keywords:** aging, hyperglycemia, cerebral blood flow, vascular smooth muscle cells, pericytes

Aging, diabetes (DM), and hypertension are leading causes of cerebrovascular diseases (CVDs), contributing to stroke and cognitive impairments and CVD-related long-term disability and death. Emerging evidence suggests that mural cells on the cerebrovascular wall play an essential role in the regulation of cerebral vascular function. However, underlying mechanisms of how vascular smooth muscle cells (VSMCs) and pericytes interact with other cerebral vascular cells to modulate the autoregulation of cerebral blood flow (CBF) and maintain blood-brain barrier (BBB) integrity in aging and DM are just beginning to be understood.

Autoregulation of CBF is impaired in aging, diabetes, and hypertension [1]. Recent studies demonstrated that VSMC contractile dysfunction is associated with poor CBF autoregulation in DM rats [2] that is exacerbated by aging [3]. Cerebral VSMCs lost contractile capability when challenged with high glucose (HG) at a similar concentration as seen in DM animal models and patients, possibly due to enhanced oxidative stress. Furthermore, HG-treated cerebral VSMCs displayed mitochondrial dysfunction and reduced ATP production [2]. These results correspond to the impaired myogenic response of the middle cerebral artery (MCA) isolated from DM rats [2,3]. Interestingly, these findings have been observed in HG-treated alpha-smooth muscle actin (α-SMA)-positive cerebral vascular pericytes in a recent study [4]. In parallel with diminished cell contractility, ATP production, and mitochondrial respiration in HG-treated pericytes, the authors also found that the myogenic response of isolated parenchymal arterioles (PAs) from DM rats was impaired. The myogenic response is the major controller of CBF autoregulation in response to elevations in systemic pressure. When cerebral VSMCs and α-SMA-positive pericytes lost the ability to constrict in response to an elevation in transluminal pressure in a hyperglycemia environment, cerebral arteries and arterioles could not protect against changes in pressure and transmitted increased pressure to fragile downstream capillaries, resulting in BBB leakage, micro- or macro-hemorrhages, neurodegeneration, and cognitive impairments [1]. This hypothesis was recently validated with *in vivo* studies that CBF autoregulation was impaired in the surface and deep cortex in elderly animal models with long-standing DM [3].

BBB dysfunction is a diagnostic and prognostic biomarker for stroke and possibly cognitive impairments [5]. At the cerebral capillary wall, the BBB is composed of endothelial cells (ECs), astrocytes, pericytes, and basement membrane. Cerebrovascular pericytes at the capillary levels often do not express α-SMA [6,7]. They are embedded in the basement membrane and crosstalk with ECs to maintain integrity of the BBB. Recent studies indicated that the thickness of the basement membrane of cerebral capillaries increased in old DM rats in association with enhanced production of advanced glycation end products (AGEs), which promoted release of transforming growth factor-β1 (TGF-β1) by pericytes and resulted in fibronectin production [4]. AGEs also evoked release of vascular endothelial growth factor (VEGF) by ECs and pericytes, which down-regulated expression of tight junction protein claudin 5. Additionally, expression of integrin-β1 in pericytes was diminished in the brain of old DM rats, which reduced the anchoring of pericytes to the basement membrane. Alterations in cerebrovascular pericyte-involved signaling pathways likely contributed to reduced pericyte and tight junction coverage, which are consistent with enhanced BBB leakage and microhemorrhages observed in old DM animal models and human studies [3,4].

Cerebrovascular pericytes are also major components at the neurovascular unit (NVU) at capillary beds. Enhanced neuronal activity at the NVU evokes CBF (functional hyperemia), independent of changes in blood pressure, that allows the blood supply to meet the metabolic demands of the brain. It involves local activation of astrocytes and mural cells by metabolic mediators and potassium ions released by neurons. The mural cells interact with capillary ECs to produce a retrograde transmitted hyperpolarizing wave via gap junctions when local neuron activity is elevated. This hyperpolarizing wave dilates α-SMA-positive pericytes at the capillary junctions and redistributes capillary flow to areas of need. It also propagates along the ECs to hyperpolarize VSMCs and increases flow in the upstream penetrating arterioles and pial arterioles [8]. Cerebral vascular dysfunction and reductions in functional hyperemic responses precede cognitive dysfunction in elderly hypertensive, diabetic, and Alzheimer's disease and Alzheimer's disease-related dementias (AD/ADRD) patients, and in old DM animal models [3]. Impairments in CBF autoregulation associated with aging and DM that alters the function of cerebrovascular mural cells can interfere with the initiation and transmission of functional hyperemic responses at many levels. Identification of the cell types and pathways involved is an area of intense investigation for the treatment of AD/ADRD associated with aging and DM.

In summary, aging exaggerates the effects of DM, and together they act as the destroyer of the cerebrovascular wall. Hyperglycemia induces dysfunction of cerebrovascular VSMCs and α-SMA-positive-pericytes due, at least partly, to its deleterious effects in enhancing oxidative stress, reducing ATP production, and diminishing contractile capabilities. The mural cell dysfunction contributes to the impaired myogenic response of cerebral arteries and arterioles and poor CBF autoregulation in the surface and deep cortex, which plays a pivotal role in the development of BBB leakage. Hyperglycemia also induces dysfunction of α-SMA- negative pericytes at the capillary levels by modulating signaling pathways involving pericyte-EC and pericyte-basement membrane crosstalk, resulting in BBB leakage by reducing pericyte and tight junction numbers. It also induces neurovascular uncoupling and disrupts functional hyperemic response. Defining the synergistic detrimental effects of aging and DM on cerebrovascular mural cells and the mechanisms involved holds tremendous promise in exploring novel drug targets for the prevention and treatment of stroke and AD/ADRD.
